# The Incidence of Atrial Fibrillation After Percutaneous Patent Foramen Ovale Closure Detected by Implantable Loop Recorders

**DOI:** 10.1016/j.jscai.2024.101930

**Published:** 2024-06-04

**Authors:** Komal Imtiaz, Mohammed Ebrahim, Jianli Niu, Jonathan Roberts

**Affiliations:** Department of Cardiovascular Disease, Memorial Healthcare System, Hollywood, Florida

**Keywords:** atrial fibrillation, cryptogenic stroke, implantable loop recorder, patent foramen ovale, percutaneous closure of patent foramen ovale

## Abstract

**Background:**

Patent foramen ovale (PFO) is seen in 25% of the general population but in up to 50% of patients ≤60 years old with cryptogenic strokes. Trials have shown that PFO closure vs medical therapy reduces the risk of future strokes. PFO closure may cause atrial fibrillation (AF), with prior trials reporting an incidence of 2% to 11.9%. However, the true incidence of AF after PFO closure is unknown due to limitations in prior studies for long-term monitoring.

**Methods:**

This is a retrospective observational study at a single center. Patients who underwent PFO closure and had an implantable loop recorder prior to PFO closure were included. The final review included 38 patients who had at least 2 months of implantable loop recorder data post-PFO closure.

**Results:**

Ten out of 38 (26%) patients developed AF post-PFO closure. The median time to the first episode of AF was 3.95 weeks, with 40% having their first AF episode after 3 months. Median duration of AF episodes was 1 hour. One hundred percent had spontaneous termination of AF. Of the AF patients, 70% were started on oral anticoagulant therapy.

**Conclusions:**

Our review shows a higher incidence of AF post-PFO closure as compared with most reported prior studies. We recommend larger prospective studies to explore the true incidence of AF post-PFO closure, its clinical impact, and subsequent stroke risk.

## Introduction

Patent foramen ovale (PFO) is present in 20% to 25% of the general population,^1^ and in most it is a benign incidental finding. In young patients (≤60 years old) with an ischemic cryptogenic stroke, the incidence of a PFO is much higher, ranging between 40% and 56%,^1^ suggesting a strong association between PFO and cryptogenic ischemic stroke in these patients. This led to multiple randomized controlled trials showing that PFO closure reduced the incidence of recurrent strokes as compared to medical therapy alone.^2^, ^3^, ^4^ Prior studies including systematic reviews have reported an incidence of atrial fibrillation (AF) after PFO closure of 2%-11.9%.^2^, ^3^, ^4^, ^5^, ^6^, ^7^ The weakness of this data was that these studies used self-reported palpitations, intermittent random electrocardiograms (ECG), or brief Holter monitors to detect AF. It is known that up to 40% of patients with AF have no specific symptoms.^8^ The true incidence of AF after PFO closure is therefore unknown, as continuous long-term implantable loop recorders (ILR) were not used in these studies post-PFO closure.

## Background

We reviewed the literature regarding PFO closure as the treatment of cryptogenic stroke and the onset of newly detected AF in this patient population. This included major randomized trials of medical therapy vs medical therapy with device closure published between 2008 to 2017 and 2 large meta-analyses and systematic reviews from 2019 and 2021.^2^, ^3^, ^4^^,^^9^, ^10^, ^11^

A comprehensive meta-analysis of 8 randomized trials of medical therapy vs PFO closure for the treatment of cryptogenic stroke included 3924 patients, and the incidence of new-onset AF was recorded.^10^ At a mean follow-up of 2.8 ± 1.7 years, the mean incidence of AF in the PFO closure group was 3.2% vs 0.47% in the medical treatment group. PFO closure was associated with a higher incidence of early-onset AF (2.9%) vs late-onset AF (0.52%). This demonstrated a 4-fold increased risk of new-onset AF when compared with control.^10^

In all the above-mentioned trials, we reviewed the protocols used for monitoring AF post-PFO closure. These included ECG in outpatient clinic appointments or outpatient Holter monitors. The REDUCE trial followed patients for 2 to 5 years and conducted outpatient visits with ECG only.^4^ The RESPECT trial performed ECG or Holter monitoring at baseline, discharge, and at a 1-month outpatient visit with a median follow-up of 5.9 years.^2^ The CLOSE PFO study performed ECG and outpatient Holter up to a median follow-up of 5 years.^3^

Ravellette et al^12^ mentioned in their review of 445 patients who underwent PFO closure an incidence of AF in 6.7% within 6 months of PFO closure. Incidence of AF, atrial flutter, and arrhythmias were assessed by ECG within 6 months from closure. This was a major limitation as the study relied on the patient’s self-reported symptoms.

None of the above-mentioned studies used ILR, so the true incidence of post-PFO closure AF is uncertain.

When PFO closure devices became FDA-approved in October 2016, our institution, Memorial Healthcare System (MHS), developed a Heart Brain Team to review all patients prior to PFO closure. This team consists of stroke neurologists, neuro-imagers, pediatric and adult interventional cardiologists, electrophysiologists, and hematologists. Upon the advice of our electrophysiologists, all patients above the age of 40 years would get an ILR and be monitored for at least 3 months, to rule out occult paroxysmal AF as an etiology of cryptogenic ischemic stroke. As a result, we have a pool of patients who had an ILR for at least 3 months prior to their PFO closure, and then for months/years following PFO closure.

We have identified a research gap in previously performed PFO trials regarding the true incidence of post-PFO closure AF. Our goal is to utilize the more rigorous AF evaluation and follow-up protocol developed by the MHS Heart Brain team to determine the true incidence of post-PFO closure AF.

## Study design and methods

This is a retrospective observational study performed at Memorial Regional Hospital, Hollywood, Florida. Patient data were collected from January 2016 through December 2021. All patients were included who had a diagnosis of cryptogenic stroke and had an ILR as a part of their cryptogenic stroke evaluation before the percutaneous closure of their PFO. Patients without an ILR prior to PFO closure or at least 2 months of post-PFO closure ILR data were excluded from the study.

Baseline patient characteristics, ILR data, and procedural details were collected via chart review of electronic healthcare records. Data were stored in a password-protected coding sheet in the institution’s secure server. ILR device type varied. ILR data were obtained by reviewing the downloaded interrogation reports (which were scanned in their electronic healthcare record) and/or cardiology office clinic notes.

Patient characteristics and outcomes are reported as frequencies and proportions for categorical variables and means with standard deviations or medians and interquartile ranges for continuous variables, as appropriate. For descriptive and outcome analyses, the study population was grouped into those with or without AF post-PFO closure. Between-group differences were evaluated using unpaired *t* tests, Mann-Whitney rank sum test, or χ^2^ tests, as appropriate. All *P* values reported are 2-tailed, and a *P* value <.05 was considered statistically significant. All statistical analyses were performed using SPSS software version 28 (IBM Corp).

We reviewed the electrophysiologists’ and cardiologists’ evaluation of the ILR data for AF detection. No interobserver variability was noted. The data were reviewed 3 times during the process of data collection and no intraobserver variability was noted.

## Results

**Figure 1:**
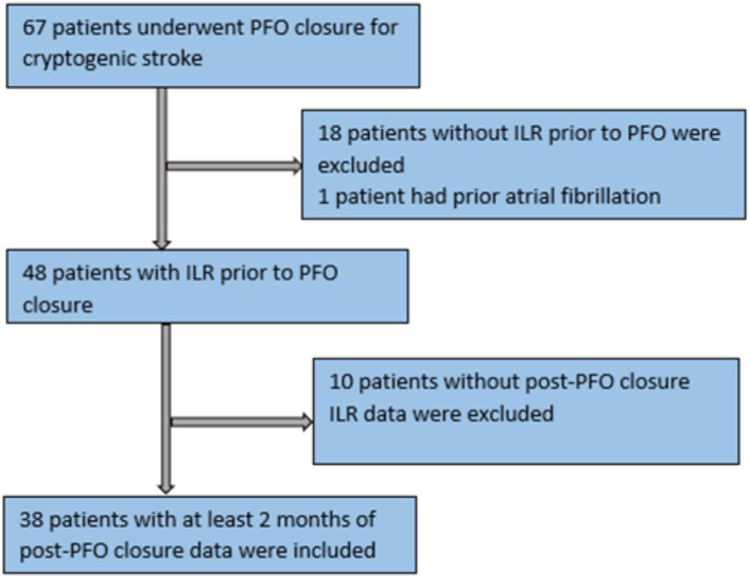
**Study flowchart.** ILR, implantable loop recorder; PFO, patent foramen ovale.

During the study timeframe, 67 patients underwent PFO closure at MHS for cryptogenic stroke. Of those 67 patients, 18 patients did not have an ILR prior to PFO closure and 1 patient had a missed diagnosis of paroxysmal AF prior to PFO closure. Forty-eight patients had an ILR for at least 1 month prior to PFO closure. Ten patients were excluded, 9 with no ILR data post-PFO closure because they were followed by an external institution and their interrogation reports could not be obtained, and 1 whose ILR was removed immediately after PFO closure at the patient’s request. Our final cohort was 38 patients who had ILR data available for at least 2 months after PFO closure (Figure 1).

The average age of patients was 53 years and 43.6% were women. The average ILR duration prior to PFO closure was 3 months and post-PFO closure was 12 months. The average risk of paradoxical embolism score was 6. All patients had an ischemic stroke based on their brain MRI findings. Both GORE CARDIOFORM Septal Occluder (81.6%) and Amplatzer Cribiform Occluder (Abbott) (18.4%) were used in the study patients, as per the interventional cardiologist’s preference. There appeared to be a higher proportion of postprocedural AF in the group that received the Amplatzer device (43%) compared to those who received a GORE device (23%); however, this was not statistically significant (*P* = .351). There was no significant association between AF incidence and size of PFO closure device (*P* = .236). There were no recurrent strokes during the monitoring period (Tables 1 and 2).

**Table 1:** Baseline characteristics of study patients.

Characteristics	Total (n = 38)	Postimplant AF (n = 10)	No postimplant AF (n = 28)	*P* value
Age, y	53.4 ± 12.2	52 ± 13.8	53.9 ± 11.7	.695
Female sex	17 (43.6)	4 (40)	13 (46.4)	>.999
Active smoking	3 (7.9)	2 (20)	1 (3.6)	.164
History of prior stroke	3 (7.9)	0 (0)	3 (10.7)	.552
Diabetes mellitus	6 (15.8)	3 (30)	3 (10.7)	.310
CHA_2_DS_2_-VASc score	3.0 (2.8-4.0)	3 (2.8-4)	3 (2.3-4.0)	.751
RoPE score	6.0 ± 1.7	6.1 ± 1.9	6.0 ± 1.7	.846
Indication of PFO closure				.263
Stroke	37 (97.4)	9 (90)	28 (100)	
Retinal artery occlusion	1 (2.6)	1 (10)	0 (0)	
Duration of ILR prior to PFO closure, mo	3 (2.0-4.0)	3 (1.5-4.5)	3 (2.3-4.0)	.676
Location of stroke^a^				
Frontal cortex	10	7	3	.001
Parietal cortex	8	5	3	.019
Temporal cortex	5	3	2	.103
Occipital cortex	4	0	4	.556
Basal ganglia	7	0	7	.156
Thalamus	4	2	2	>.999
Cerebellum	10	3	7	>.999
Brain stem	2	0	2	>.999
Insular cortex	3	0	3	.552

Data are given as mean ± SD, median (IQR), or n (%).

ILR, implanted loop recorders; PFO, patent foramen ovale; RoPE, risk of paradoxical embolism.

a Location of stroke: n represents the total number of stroke lesions per location as seen on brain imaging. Some patients had multiple strokes in different anatomical locations.

**Table 2:** Procedural characteristics of study patients.

Variable	Total (n = 38)	Postimplant AF (n = 10)	No Postimplant AF (n = 28)	*P* value
PFO closure devices				.351
GORE CARDIOFORM Septal Occluder	31 (81.6)	7 (70)	24 (85.7)	
AMPLATZER Cribriform Occluder	7 (18.4)	3 (30)	4 (14.3)	
Size of PFO closure devices used				.236
25 mm	28 (73.7)	9 (90)	19 (67.9)	
30 mm	10 (26.3)	1 (10)	9 (32.1)	
Procedural complications				—
Pericardial effusion	0 (0)	0 (0)	0 (0)	
DVT post-PFO closure	0 (0)	0 (0)	0 (0)	
Sustained AF during PFO closure	0 (0)	0 (0)	0 (0)	
Initial postprocedural antithrombotic therapy				.235
Aspirin	36 (94.7)	8 (80)	28 (100)	
P2Y12 inhibitor	36 (94.7)	9 (90)	27 (96.4)	
DOAC	3 (7.9)	2 (20)	1 (3.6)	
Duration of ILR post-PFO closure, mo	12 (6-12)	12 (7.5-12)	12 (6-12)	.720

Data are given as median (IQR) or n (%).

AF, atrial fibrillation; DOAC, direct-acting oral anticoagulant; DVT, deep vein thrombosis; ILR, implanted loop recorders; PFO, patent foramen ovale.

**Figure 2:**
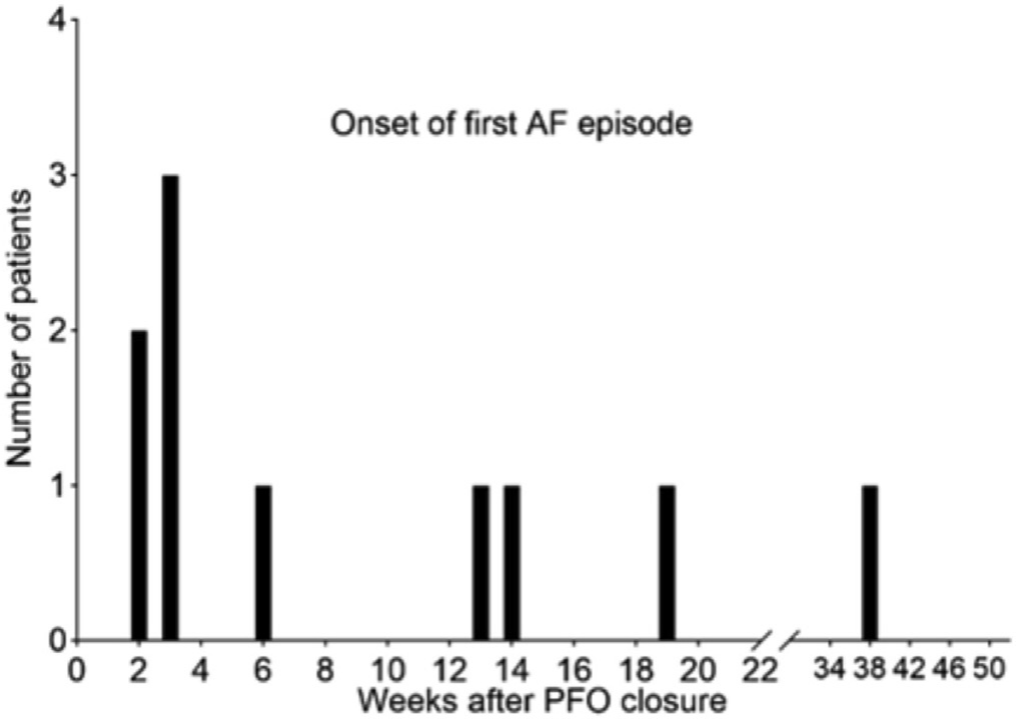
**Timing of first atrial fibrillation (AF) episode by week after patent foramen ovale (PFO) closure**.

**Figure 3:**
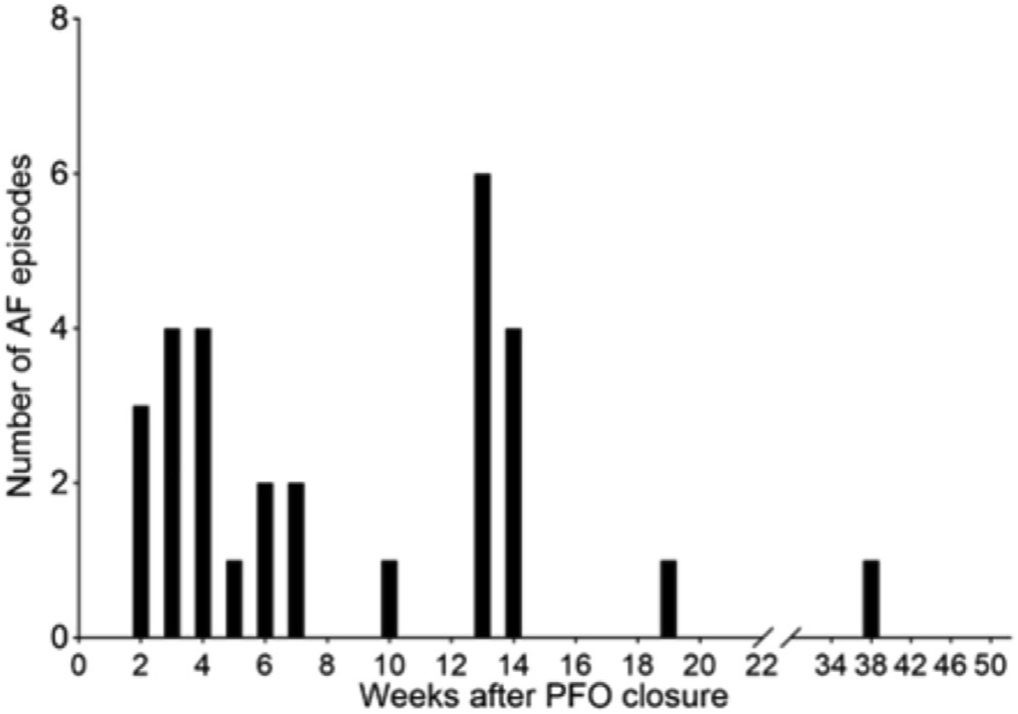
**Total number of atrial fibrillation (AF) episodes that occurred by weeks after patent foramen ovale (PFO) closure**.

Ten out of 38 (26%) patients developed AF post-PFO closure. The median time to the first episode of AF was 3.95 weeks, with 40% having their first AF episode after 12 weeks (Figures 2 and 3). Median duration of AF episodes was 1 hour (Table 3). One hundred percent had spontaneous termination of AF and did not require rate control therapies (calcium channel or beta blockers) and/or antiarrhythmic agents. Of the AF patients, 70% were started on oral anticoagulant therapy (Table 3) by the judgment of their clinical cardiologists and not by a specific institutional protocol.

**Table 3:** Treatment characteristics of patients with postimplant AF.

Treatment variable	Patients with postimplant AF (n = 10)
Time to first episode of AF, wk	3.95
Duration of AF, h	1.0 (0.21-13.68)
Spontaneous termination of arrhythmia	10 (100)
Initiated on beta blocker	0 (0)
Initiated on calcium channel blocker	0 (0)
Initiated on other antiarrhythmic therapy	0 (0)
Direct current cardioversion	0 (0)
Transitioned to anticoagulation for PIAF	
Direct-acting oral anticoagulant	7 (70)

Data are given as median (IQR) or n (%).

AF, atrial fibrillation; DOAC, direct-acting oral anticoagulant; N/A, data not available; PFO, patent foramen ovale closure; PIAF, postimplant atrial fibrillation.

## Discussion

The most important finding of our study was the high incidence of post-PFO closure AF (26%), compared with prior studies reporting an incidence of 2%-11.9% (Central Illustration).^2^, ^3^, ^4^, ^5^, ^6^, ^7^ We believe this is likely because of our use of ILR in all our patients. In our study, the mean time to onset of AF post-PFO closure was 4 weeks, with 40% having their first episode at 3 months or later. Is this high incidence of post-PFO closure AF of clinical significance, ie, do these patients have a higher incidence of stroke recurrence, and would they benefit from prolonged oral anticoagulation therapy? The small sample size and lack of prolonged clinical follow-up in our study do not allow an answer to these important questions.

**Central Illustration:**
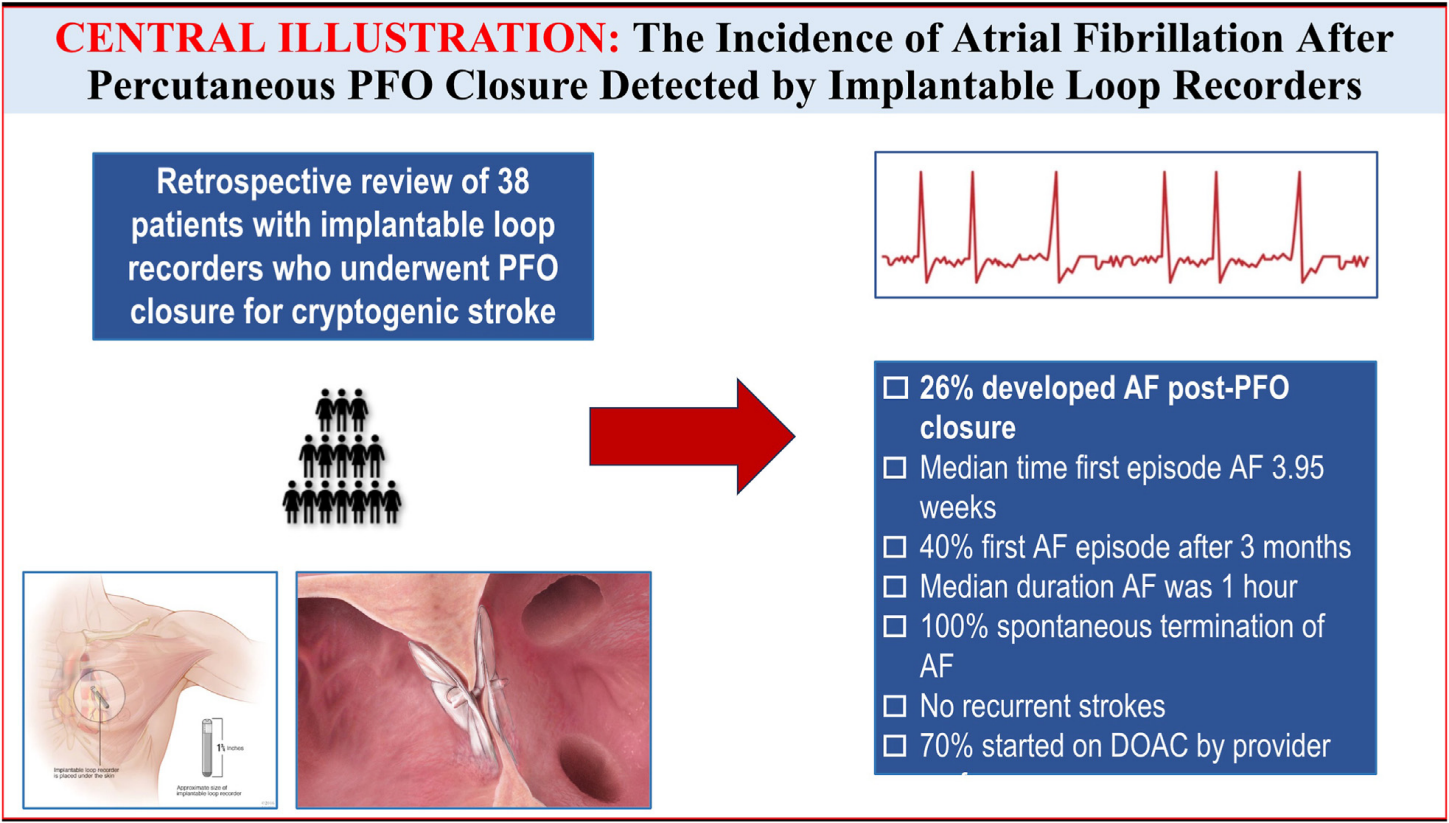
The incidence of atrial fibrillation (AF) after percutaneous patent foramen ovale (PFO) closure detected by implantable loop recorders. DOAC, direct oral anticoagulation.

As our study was a retrospective trial, we only reported how individual clinicians decided to treat the patients based on their clinical judgment. Whether direct oral anticoagulation was clinically beneficial or harmful to these patients is unknown.

One of the strengths of our study is robust long-term data from ILR in our patients who underwent PFO closure, which allowed the detection of even asymptomatic AF episodes. Our devices were set to detect episodes of AF with a duration of at least 2 minutes as per the ILR AF detection algorithms.

Our findings were concordant with the higher incidence of AF post-PFO closure (37%) reported in the study by Krishnamurthy et al.^13^ In their study, they had a similar sample size of 35 patients who had pre-PFO closure loop recorders. They reported that most episodes of AF occurred within the first 4 weeks. In our study, as well as in the study of Krishnamurthy et al,^13^ no cases of persistent AF were observed. Krishnamurthy et al^13^ also reported an older age of those who developed post-PFO AF vs those who did not (mean 62 years vs 52 years), which we did not observe in our study (mean 52 years vs 54 years).

Most recently, Guedeney et al^14^ published a retrospective review of AF detection after PFO closure using loop recorders in 225 patients. AF was detected in 13 (9.9%) and 24 (28.9%) patients monitored with 4-week external loop recorders or implantable loop recorders, respectively. This study also documented a higher incidence of AF post-PFO closure when ILR were used compared with most previous studies of AF post-PFO closure detected by patient symptoms and/or intermittent ECG or external monitors.

It may be important to distinguish traditional spontaneous AF from post-PFO closure AF, both mechanistically and regarding stroke implications. Traditional spontaneous AF is usually characterized by rapid atrial firing that triggers AF in the pulmonary veins.^15^ Potential mechanisms of AF after PFO closure include local stretch and irritation from the device itself. The device may also lead to a local inflammatory response that can trigger atrial arrhythmias.^1^, ^5^ These post-PFO closure inflammatory and local stretch factors likely “heal” weeks to months after the device is implanted and may account for the resolution of AF post-PFO closure after several months in most patients and the self-terminating nature of AF post-PFO closure.

Our small study did not observe any stroke recurrence in patients with AF post-PFO closure, similar to the larger study of 445 patients reported by Ravellette et al.^12^ It has been reported that higher burden AF has been related to increased stroke risk.^16^ Could the small burden of AF post-PFO closure, ie, a median of 1 hour in our study with 100% spontaneous termination, make postprocedural AF less likely to cause thromboembolic strokes than traditional spontaneous AF? Could there be a corollary to the low burden of subclinical AF of <24 hours duration having an equal thromboembolic risk to persons without AF as found in the recent ASSERT trial?^17^ Our current knowledge base of AF post-PFO closure does not allow an answer to the important question of whether oral anticoagulation therapy should be prescribed to those patients who have postprocedural AF, to possibly reduce the chance of a thromboembolic stroke. Is the AF seen on ILR post-PFO closure actionable data or just noise? Future larger trials will be needed to answer this question.

Limitations of our study include its retrospective design, small sample size, limited follow-up period, and difficulty in attaining medical/loop records in some patients. The detection algorithm of the loop recorders is by no means infallible, but we made a concerted effort to review every detected episode to ensure accurate identification of AF.

## Conclusion

Our study showed a much higher incidence of post-PFO closure AF (26%) than seen in multiple prior larger studies (2%-11.9%) that did not use ILR for AF detection. AF episode duration in our study averaged 60 minutes, 100% self-terminated, and most occurred within weeks after PFO closure. Are there selected patients with post-PFO closure AF who may benefit from oral anticoagulation or is it just needlessly adding the bleeding risks of anticoagulation? Larger prospective studies will be needed to determine the true incidence of post-PFO closure AF and its clinical significance.
